# Road Traffic Noise Exposure in Gothenburg 1975–2010

**DOI:** 10.1371/journal.pone.0155328

**Published:** 2016-05-12

**Authors:** Mikael Ögren, Lars Barregard

**Affiliations:** Occupational and Environmental Medicine, Institute of Medicine, Sahlgrenska Academy, University of Gothenburg, Gothenburg, Sweden; Boston University, UNITED STATES

## Abstract

Traffic noise exposure within a city varies over time and space. In this study, we developed a modified noise calculation method and used this method together with population and traffic data to estimate the time trend of noise exposure for the population in Gothenburg, Sweden, from 1975 to 2010. The noise calculation method was based on the standard Nordic method for road traffic noise with modifications using area-level statistics for population and building structures instead of precise geocoding of each inhabitant. Noise emission per vehicle was assumed to be constant over the period. The results show an increase in noise exposure over time. The number of inhabitants exposed at an equivalent level above 55 dB increased from 93000 to 146000 inhabitants between 1975 and 2010, and the percentage of the population exposed at this level increased from 22% to 29% over the same period. Traffic increase (1.4% per year) and population increase/concentration (0.50% per year) were approximately equally important factors behind this increase in exposure.

## Introduction

Noise exposure from road traffic can be assessed by direct measurements when the focus is on limited populations and time periods; but for larger populations and longer periods, calculations are performed using validated models, usually at the façades of the dwellings using data on noise emission per vehicle, traffic flows, residential buildings, and terrain.

For public health reasons it is of interest to follow up noise exposure over time, and longitudinal epidemiological studies of the health effects of noise often need to estimate noise exposure in the past, many years before the health outcomes of interest. Performing such longitudinal studies in large areas is a difficult task, and few studies have been published. In [1] the noise exposure at 62 000 adresses in Cophangen was estimated for 1990, 1995, 2000 and 2005. Another paper compared the noise levels in the central parts of Amsterdam in 1930 and 2010 [2].

There is an ongoing effort to follow the time trend of noise exposure in Europe via the Environmental Noise Directive (END), which requires that all member states of the European Union periodically report the result of a strategic noise mapping for major cities, roads, railways and airports [3]. The noise mapping is to be performed every five years starting from 2006, and some results are available on-line for 2006 and 2011 [4]. Preliminary analyses indicate that the exposure has increased in areas where maps are available for both periods [5], but it is hard to assess an overall trend since much of the data has not yet been reported by the member states.

Obtaining input data of sufficient quality for all necessary parameters is a major concern when mapping noise exposure, particularly when trying to map this exposure backwards in time. Moreover, for large areas the calculations can take a long time even with modern high-capacity computers. For these two reasons it is useful to modify existing calculation methods in order to obtain good-quality results with less detailed input data and fewer computational resources.

One example of such a simplified method is the TRANEX method [6], which is based on the British standard calculation method [7] but has been modified to facilitate large-scale noise mapping. The method does not require the precise location of the receiving points and terrain heights, but does require building outlines. When compared against a large set of measurements (equivalent level over 30 minutes) at two different sites the results appeared promising, with a Spearman correlation coefficient of 0.90 for predicted and measured values. The method overestimated the noise level in areas with low noise levels, but performs better at high noise level. The root-mean-squared error was 3.1 dB.

The aim of the present study was to investigate the time trend of residential noise exposure in Gothenburg, Sweden from 1975 to 2010 using traffic flow measurements and population statistics. The method used is based on the Nordic method for road traffic noise [8], modified to approximate the effect of multiple reflections between building façades and to remove the need for precise information on buildings and receiver locations.

## Methods

### Ethics statement

No specific ethic permissions were needed for this study. The main result is based on area level data (grid size 100 m), and no geocoded addresses or other data on individuals were collected or used. For the smaller test areas used in section no data on individual inhabitants were used, only the location and the total number of inhabitants of each dwelling were recorded. The study design was audited by Statistics Sweden and found to comply with their recommendations regarding use of population data before the study was started.

### Traffic flow and noise emission

Our estimates of traffic flows were based on a geographic information system (GIS) data set prepared for the Gothenburg official END noise map for 2006. Gothenburg is a city on the west coast of Sweden with approximately 550 000 inhabitants as of 2010. The dataset contains a GIS road network with road traffic flows, maximum allowed speeds and percentages of heavy vehicles. The traffic data was estimated from traffic measurements and traffic flow models for the majority of road links. A default traffic flow was set for roads where no measurements or traffic model predictions were available according to a scheme based on the official road classification [9]. The absolute minimum traffic flow for small streets was set at 250 vehicles per 24 hours.

The percentage of heavy traffic was also coded for each road link; this was based on measurements where available, but for most links only the total traffic flow was measured. In these cases other parameters such as maximum allowed speed, road classification and road width were used to manually assign the percentage of heavyy vehicles [9].

In the present study the GIS dataset with traffic flows for 2006 was expanded to a yearly estimate for the period 1975–2010, using a database of traffic flow measurements starting from 1975. Most measurements was performed with pneumatic tubes across the road during one or more days, but some utilized electronic sensors. The measurements were then used to estimate the yearly average vehicle flow for 24 hours at the measurement point. This resulted in a database of approximately 16,000 measured traffic flows distributed over different positions and over time from 1975 to 2010.

**Fig 1:**
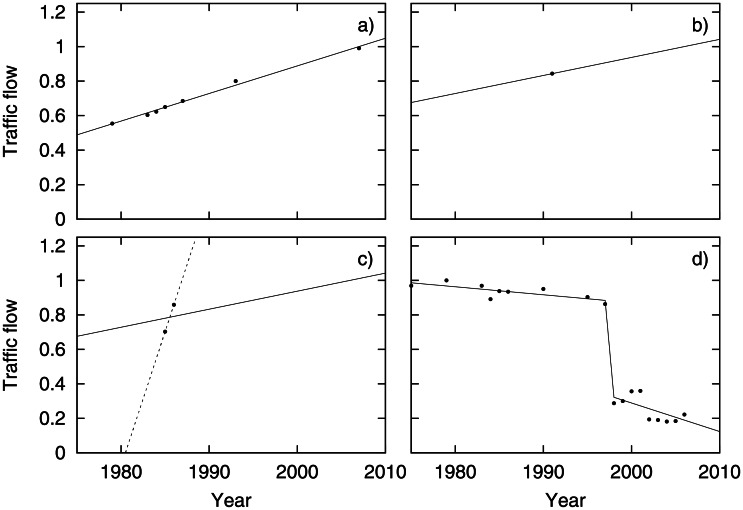
Illustration of estimation strategy for time trend of traffic flow.

In order to estimate the traffic flow over time for each link the following strategy was used. For each road link a piece-wise linear function with one or two breaking points for the period was estimated from the measurements, see Fig 1. The following categories of measurement configurations were used to estimate the time trend (illustrated by the sub-figures in Fig 1):

1. Using linear regression for the complete period.
2. Using the average slope over all measurements if only one measurement was available.
3. Using the average slope if only two or three points with little spread over time were available, in order to avoid extreme traffic estimates when extrapolating (see the dotted line).
4. Using a piecewise function when major traffic changes had occurred during the period, including closing old road links or opening new ones.

All adjustments using methods c) and d) were manually introduced by visual inspection of the regression graphs. Most of the manual adjustments were needed due to road links being built or removed during the period of interest, especially around the three major tunnel projects in Gothenburg during this time: Gnistängstunneln in 1978, Lundbytunneln in 1997 and Götatunneln in 2006.

### Prediction of noise levels

The noise levels were estimated with the Nordic method for road traffic noise prediction [8], which calculates a sound power level from the traffic flow, percentage of heavy vehicles, and speed. The sound pressure level at the receiver is then calculated as the sum of the contributions from all roads with adjustments for propagation distance, screening by terrain/barriers and the ground effect (the effect of propagation close to acoustically soft ground).

For a typical noise calculation, the receiver position is located on a building façade that corresponds to a dwelling. If the dwelling has access to several façades of the building, for example a detached house or an apartment with several rooms connected to different façades, the noise level is normally calculated at the most exposed façade. In this simplified strategic noise mapping procedure, no information on the precise location of buildings or inhabitants is available, so instead every point on the map is seen as a potential location of the most exposed façade of a building.

In dense urban areas, it is important to include the effect of acoustic reflections from building façades, especially in urban canyon situations; that is, when a street is surrounded by more or less continuous building façades on both sides. When using the Nordic method and including reflections up to the third order, which is fairly typical for detailed calculations, the levels at the façade in urban canyons are 3–6 dB higher than for streets with buildings on one side only [10]. More detailed calculations [11, 12] and field measurements [13] show similar results, the most important parameter being the acoustical absorption on the surfaces of the canyon. Since no information on the precise location of buildings was available in our simplified input data, areas with a street canyon building structure were manually marked, and an adjustment of +4 dB was applied for positions within 25 m of the road center line, corresponding to the exposed façades along the street canyon.

The height of the receiver above ground is an important parameter in noise calculations, especially where the source is far from the receiver or where screening by terrain or buildings causes the higher floors of a building to be more directly exposed. In this study, no information on the height above ground level of the exposed population was available, so nominally a height of 2 m above ground level was used, which is typical for large scale noise maps in Sweden [14, 15].

The Nordic method was revised in 1996, and the noise emission coefficients are valid for vehicles in traffic at that time. Research results from the Nordic countries [16] indicate that the average noise emissions of heavy vehicles remained approximately unchanged between 1970 and 2005. Light vehicles show a small increasing trend during the same period, but since there are many uncertainties regarding the exact details of what is causing the observed change we decided to assume no change in emission per vehicle for both heavy and light vehicles from 1975 to 2010.

### Noise exposure

The number of inhabitants in Gothenburg over time was obtained from Statistics Sweden in a grid pattern of squares with 100 m sides. For each square the total number of inhabitants was given every fifth year from 1975 to 2010. The precise location of each inhabitant within the square was not known, and so the following method was used to estimate the positions of each inhabitant.

For each population square an area defined by all points closer to any road line than 15 m was removed, and then the population was assumed to be evenly distributed over the remaining area of the square. Note that for some squares with many road links all of the square might be removed, but in our dataset no square with a population was completely removed.

The number of exposed inhabitants exposed was estimated by summing up the integral of the noise level multiplied by the population density over the surface of each square according to

$$\begin{array}{c} M\left(L_{limit}\right)=\sum_{n=1}^{N}\;\int \;\;\;\int \left(\sigma \left(n\right)\;\theta \left(L-L_{limit}\right)\right)dxdy, \end{array}$$ (1),

where *M* is the number of inhabitants exposed at levels equal to or higher than *L*_*limit*_, *σ*(*n*) is the population density at square *n*, *L* is the equivalent level over 24 hours calculated with the simplified scheme discussed above, *θ* is the Heaviside step function and *N* is the total number of inhabited squares. The map coordinates are represented by *x* (easting) and *y* (northing). The numerical integration is solved with a basic rectangle method using a minimum of 16 rectangles, which translates to at least one evaluation of the noise level for every 25 × 25m^2^. Note that in all figures and tables below the number of exposed individuals are the total number exposed above the limit, not the number exposed between a lower and upper limit. The number of exposed above 55 dB does therefore include also those exposed above 60 dB and 65 dB.

## Results and Discussion

### Accuracy of predictions

The main result in the calculations presented here is the number of inhabitants exposed over a certain limit. Two test areas were selected to test the accuracy of this output. In these areas detailed noise maps were used to calculate the number of exposed inhabitants and the results were compared to the modified method (Table 1). The detailed noise maps were calculated using the complete Nordic method [8]. Reflections up to third order were included, and the correct height above the ground was used for the receivers. The ground effect, screening by buildings, sound barriers and terrain were also modeled as prescribed in the method. For each dwelling noise levels were calculated for all façades, and the highest level from all façades was selected as the exposure for all inhabitants in that dwelling.

**Table 1:** Number of persons in different exposure categories in two test areas, calculated both with the standard Nordic method using full information and the proposed modified method using limited information.

	Total population	Population exposed above level [dB]		
		≥ 55	≥ 60	≥ 65
Sparse area, 1–2 floor buildings				
Full method [8]	367	185	127	17
		50%	35%	4.6%
Proposed modified method		191	96	19
		52%	26%	5.1%
Dense area, 5–8 floor buildings				
Full method [8]	718	712	489	34
		99%	68%	4.7%
Proposed modified method		718	436	11
		100%	61%	1.5%
Both areas				
Full method [8]	1085	897	616	51
		83%	57%	4.7%
Proposed modified method		909	532	30
		84%	49%	2.8%

The modified method used information on the number of inhabitants in 100 m squares only (as in the dataset for Gothenburg). In a radius of 1 km around the receivers all roads were included as sources as described above. The two test areas were choosen to represent two different city landscapes: an area with separate houses and a street canyon structure with enclosed courtyards. In both cases the noise level for the year 2006 was modeled since this was the reference year for the traffic model.

The more sparsely populated area consisted of 17 squares with 100 m sides, giving a total area of 0.17 km^2^. It included 114 dwellings and a total population of 367. Most of the dwellings were detached houses with one or two floors. Two major and several minor roads crossed the area, with posted speed limits of 50 km/h or lower, and with traffic including 5–8% heavy vehicles. The traffic flows of the major roads were between 3000 and 7000 vehicles per day depending on position.

The densely populated area consisted of two squares (0.02 km^2^), and the buildings formed a street canyon structure with buildings of 5–8 floors on each side. The total population was 718, and the traffic flow varied from 21000 vehicles per day on the major roads down to the default traffic for minor roads (250 vehicles per day). The posted speed limit throughout the area was 50 km/h and traffic included 3–5% of heavy vehicles.

The results of the calculations in terms of the number of inhabitants exposed over 55, 60 and 65 dB equivalent level are presented in Table 1. The modified method predicted the number exposed above 55 dB with good accuracy. For higher levels where the number exposed was lower the accuracy was also lower, resulting in an underestimation of the number of inhabitants exposed above 60 and 65 dB.

Another way of testing the accuracy is to compare the results of the modified method to the official END noise map of Gothenburg for 2006 [9]. Note that this map uses the European noise indicator level day-evening-night (*L*_*den*_), which is an equivalent level but with increased weight for evening and night-time traffic. For typical Swedish road traffic time distributions *L*_*den*_ is 3 dB higher than the 24-hour equivalent level [17]. The comparison for the city of Gothenburg is given in Table 2 with the number of inhabitants in each category rounded to the closest multiple of 100. The modified method underestimated the number of exposed inhabitants over *L*_*den*_ 55 dB.

**Table 2:** Number of persons exposed above 55, 60 and 65 dB *L*_*den*_, calculated from the official Environmental Noise Directive (END) noise map of Gothenburg compared to calculations using the modified method.

	Population exposed above level [dB]		
	≥ 55	≥ 60	≥ 65
Gothenburg END map	97400	60400	39400
	20%	13%	8.2%
Modified method	86700	64900	30700
	18%	14%	6.4%

The official END noise exposure values are, however, not predicted with complete knowledge of every dwelling in the city, but instead use population statistics on the property level. A single property often has several dwellings, and in the denser areas of the city may even include several buildings with multiple dwellings in each building. The official END noise exposure assessment uses a simplified approach that leads to an overestimation of the number exposed in such cases. Recent unpublished estimates shows approximately 32 000 inhabitants exposed above *L*_*den*_ 65 dB, which is much closer to the modified method estimation for the exposure stratum above 65 dB.

### Time trend of noise exposure

**Fig 2:**
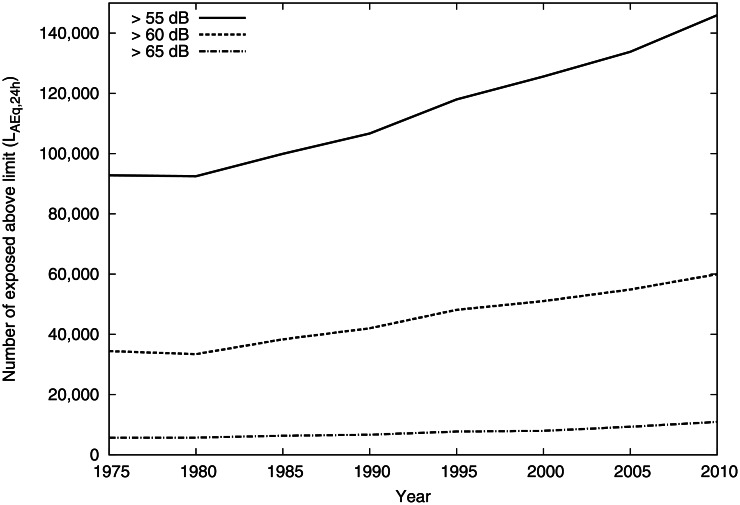
Time trend of number of individuals exposed above equivalent level 55, 60 and 65 dB.

By combining traffic estimates and population statistics with the noise exposure prediction method described above, it was possible to predict the noise exposure in Gothenburg every fifth year from 1975 to 2010. The estimated numbers of inhabitants exposed over the 55, 60, and 65 dB equivalent levels are given in Fig 2 and Table 3. In order to investigate whether population growth and relocation or traffic increase was the most important explanation, calculations were also performed first with a constant population (assuming no population increase or redistribution), and with the traffic flows from 1975 unchanged over time.

**Table 3:** Number of persons exposed above 55, 60 and 65 dB equivalent level for 1975 and 2010.

	1975	2010
≥ 55	92800	145900
≥ 60	34400	59900
≥ 65	5600	10900

**Fig 3:**
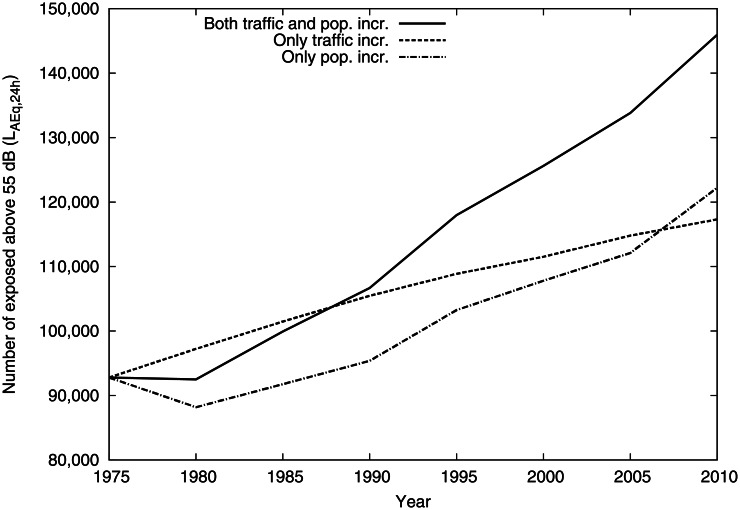
Time trend of number of exposed individual above equivalent level 55 dB comparing the default calculation to no traffic change and no population change.

The numbers of exposed inhabitants are presented in Fig 3. Using this analysis it is evident that the traffic change was the most important over the whole period, but the importance of the population increase grew (the slope of that curve is higher from about 1980 onwards). Note that population increase here also includes redistribution of population within the city.

The number of inhabitants exposed above 55 dB increased from 93 000 in 1975 to 146 000 in 2010, which translates to a yearly increase of 1.3% over the whole period. The yearly increase in population over the whole period was about 0.50%, with an initial decline in the beginning and then a steady increase of about 0.77% per year from 1985. Traffic flows increased by 1.4% per year.

In a linear regression model, based on the five-year interval data in Fig 3, the slope of the increase in number of individuals exposed above 55 dB after 1975 was 745 (95% confidence interval 701–789) per year for the increase in traffic and 628 (95% confidence interval 404–853) per year for the increase in residences. In additional statistical analyses there were no statistically significant difference in the impact of the two factors, but the comparison was based on a limited number of five-year interval values (data not shown).

Not only did the noise exposure increase in all exposure categories, but the increase was higher for the higher exposure categories. This is explained by the fact that the population increase was higher in areas with high traffic flows and higher noise levels. Most of the new dwellings were built in already dense areas, or in new areas located close to the major transport corridors. During the study period the city of Gothenburg expressed a political ambition to decrease the number of dwellings with high noise levels at the façade, and national regulations were put in place to influence city planning in the direction of reducing noise levels at the exposed façade. However, no effects of these incentives were visible in our calculations.

In order to test if our method is sensitive enough to show decreases in the number of exposed we extracted results for an area where the traffic decreased due to the opening of a new tunnel in 1997 (the Lundby tunnel). A lot of the traffic passing through the area chose the new and more time efficient route through the tunnel after 1997. Traffic flow along the main street through the area was reduced by almost 80% between 1995 and 2000, but was unchanged for many smaller local roads. The measured equivalent noise levels decreased by 6–8 dB for the most exposed buildings. In 1995 the population in the area was 128 persons, and it increased to 143 in 2000. The number of exposed above 55 dB did decrease from 93 (72%) in 1995 to 54 (38%) in 2000, which shows that the model using our input data on traffic and population did capture the decrease in the number of exposed as expected in this area.

To investigate where in the city the changes were most important we created a map of the number of inhabitants per km^2^ exposed above 55 dB equivalent level for every year. To be useful, such a map must be spatially averaged, and so we choose to use a geographically-weighted mean approach with a Gaussian kernel function of the form

$$\begin{array}{c} w\left(x,y\right)=exp(-\frac{x^{2}+y^{2}}{2\;h^{2}}). \end{array}$$ (2)

**Fig 4:**
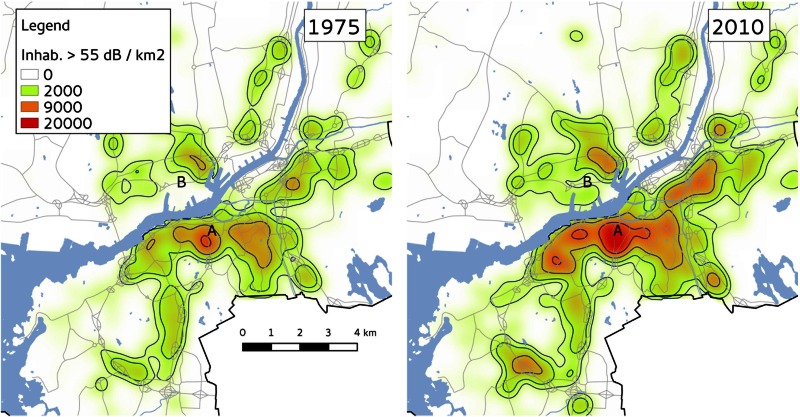
Map of the number of persons exposed above equivalent level 55 dB per square kilometer. Geometrically avaraged with a Gaussian kernel. Road lines and water boundaries obtained from Lantmäteriet (agreement number l2014/00696).

. The bandwidth parameter *h* was set to 250 m, which translates to a smoothing of the map with 90% of the weight within a distance of 530 m [18]. Fig 4 presents the map for 1975 and 2010 side by side. The largest changes are visible close to the city center and in the harbor area north of the river (marked “A” and “B” on the map). The harbour area was mainly an industrial shipyard in 1975, but has since seen the construction of many new residential buildings and an overall reduction in industrial activity.

### Discussion

This study was limited to the noise exposure from road traffic in terms of outdoor façade levels, and was thus unable to capture any effect of better sound insulation of new buildings. Future research will perhaps show how much lower noise levels are in new buildings, and what impact this changed indoor exposure might have on well-being and health. However, in terms of annoyance there is some data already available. During 1999 a national survey of the environmental impact on health was conducted in Sweden which included questions on annoyance from road traffic. When the survey was repeated again in 2007, the percentage of annoyed from road traffic noise increased from 7.9% to 11%. This is the converse of what one might have expected if new houses with better sound insulation properties had in fact an impact in terms of reducing annoyance.

**Fig 5:**
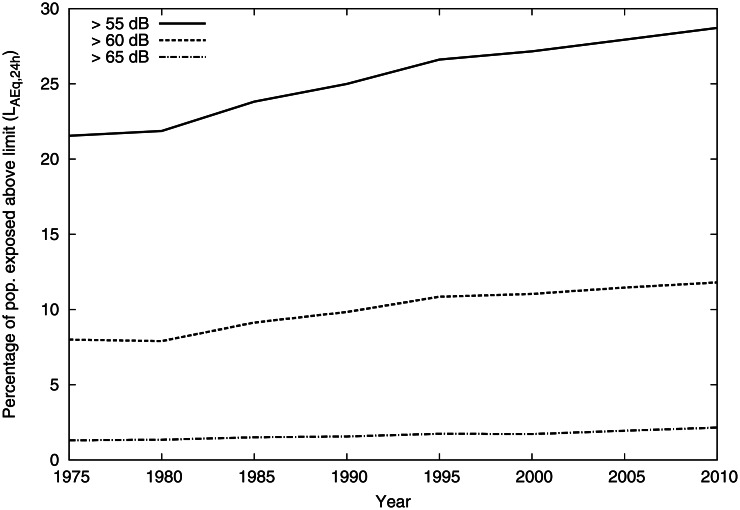
Time trend of percentage of population exposed above equivalent level 55, 60 and 65 dB.

The percentage of the population in Gothenburg exposed at the façade to an equivalent level of 55 dB or higher has increased from 21.5% in 1975 to 28.7% in 2010, see Fig 5. If the population continues to increase evenly across the city there will be no change in this percentage since all exposure groups would increase in the same manner. The potential effect due to the traffic increase is more difficult to judge. If the traffic increase occurs in areas with low population density the effect will be minimal, but if it occurs in densely populated areas, it will increases the percentage of the population exposed at higher levels.

In summary, we examined long-term time trends (1975–2010) of residential noise exposure in the population of Gothenburg. For noise calculations in large cities over long time periods modifications are necessary to decrease the requirements in manpower and computing resources. We therefore performed calculations using a modification of the Nordic method which could be run without data on the exact position of the residential addresses. Our results show that the prevalence of noise levels exceeding 55 dB at the façade has increased, due to increased road traffic and to an increasing concentration of residences close to busy roads. The two factors seem to have been approximately equally important for the increased noise exposure on a population level.

## Supporting Information

S1 Animation Animation of the number of persons exposed above equivalent level 55 dB per square kilometer from 1975 to 2010. Geometrically avaraged with a Gaussian kernel. Road lines and water boundaries obtained from Lantmäteriet (agreement number l2014/00696).
(GIF)

S1 Dataset Minimal GIS dataset. Shape files containing estimated road traffic flows, percentage of heavy vehicles, posted speed limits, noise barriers and areas considered as urban canyons for the period 1975–2010.
(GZ) Click here for additional data file.
