# Supplementary figures and images for: Road Traffic Noise Exposure in Gothenburg 1975–2010
Source: PLoS One. 2016 May 12;11(5):e0155328. doi: 10.1371/journal.pone.0155328 (PMC4865157; doi:10.1371/journal.pone.0155328)

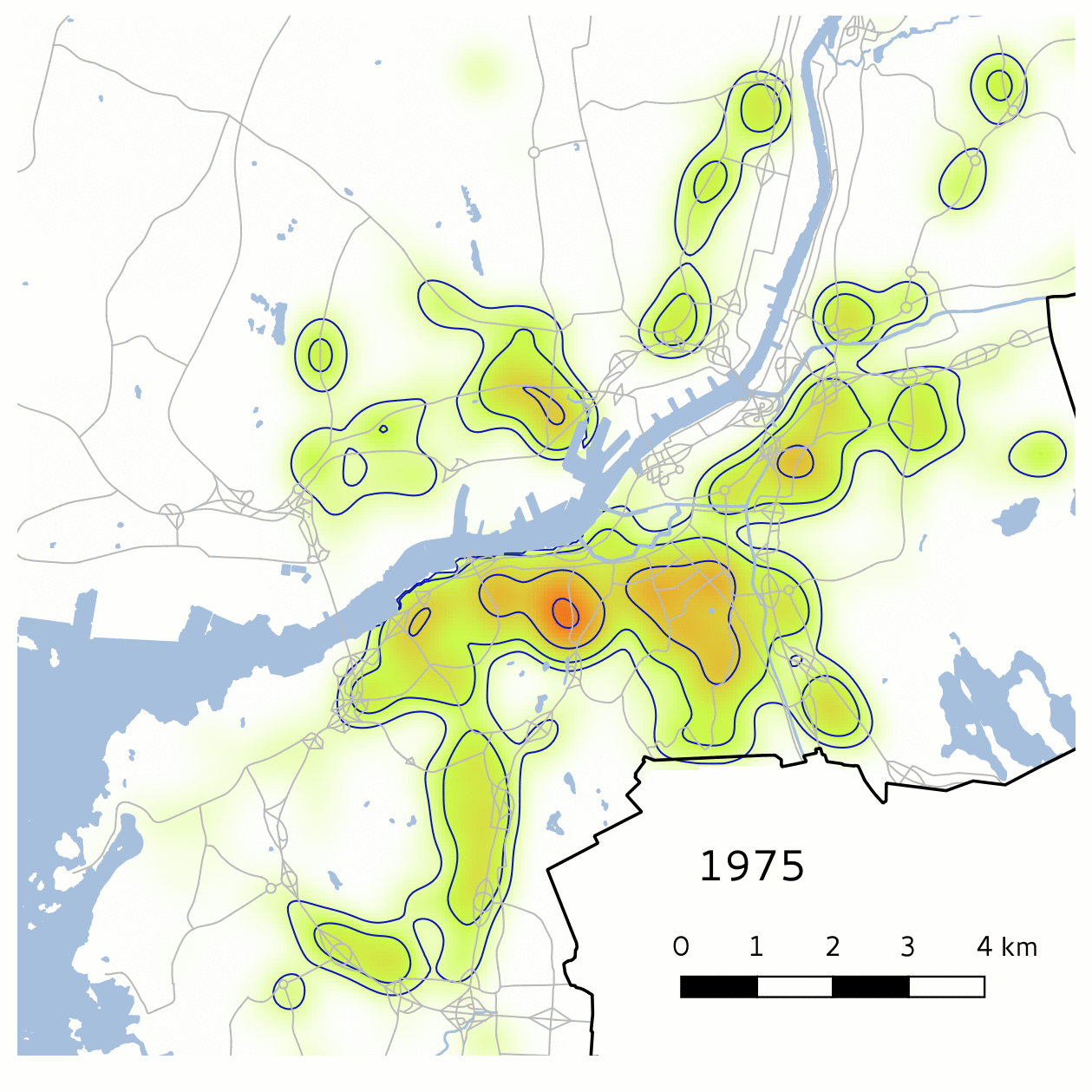

Supplement: S1 Animation — Geometrically avaraged with a Gaussian kernel. Road lines and water boundaries obtained from Lantmäteriet (agreement number l2014/00696). (GIF) [file pone.0155328.s001.gif]
